# Efficient Electrocatalytic Ammonia Synthesis via Theoretical Screening of Titanate Nanosheet-Supported Single-Atom Catalysts

**DOI:** 10.3390/ma17102239

**Published:** 2024-05-09

**Authors:** Kaiheng Zhao, Jingnan Wang, Yongan Yang, Xi Wang

**Affiliations:** 1 Institute of Molecular Plus, School of Chemical Engineering and Technology, Tianjin University, Tianjin 300072, China; kh_zhao@163.com (K.Z.); wjn10081711@163.com (J.W.); 2 Key Laboratory of Luminescence and Optical Information, Ministry of Education, School of Physical Science and Engineering, Beijing Jiaotong University, Beijing 100044, China; 3 Tangshan Research Institute of Beijing Jiaotong University, Tangshan 063000, China

**Keywords:** NRR, single-atom catalysts, theoretical screening

## Abstract

The electrocatalytic nitrogen reduction reaction (NRR) for synthesizing ammonia holds promise as an alternative to the traditional high-energy-consuming Haber–Bosch method. Rational and accurate catalyst design is needed to overcome the challenge of activating N_2_ and to suppress the competitive hydrogen evolution reaction (HER). Single-atom catalysts have garnered widespread attention due to their 100% atom utilization efficiency and unique catalytic performance. In this context, we constructed theoretical models of metal single-atom catalysts supported on titanate nanosheets (M-TiNS). Initially, density functional theory (DFT) was employed to screen 12 single-atom catalysts for NRR- and HER-related barriers, leading to the identification of the theoretically optimal NRR catalyst, Ru-TiNS. Subsequently, experimental synthesis of the Ru-TiNS single-atom catalyst was successfully achieved, exhibiting excellent performance in catalyzing NRR, with the highest NH_3_ yield rate reaching 15.19 μmol mg_cat_^−1^ h^−1^ and a Faradaic efficiency (FE) of 15.3%. The combination of experimental results and theoretical calculations demonstrated the efficient catalytic ability of Ru sites, validating the effectiveness of the constructed theoretical screening process and providing a theoretical foundation for the design of efficient NRR catalysts.

## 1. Introduction

Hydrogen (H_2_), serving as a clean energy carrier, exhibits high efficiency and zero emissions, rendering it an ideal energy substance [1,2]. However, the challenge of hydrogen storage severely restricts its applications [3,4,5]. Concurrently, ammonia (NH_3_), functioning as a hydrogen source, boasts a higher hydrogen density (contains 17.8 wt% H), implying that it can store the same quantity of hydrogen in a smaller volume [6,7,8]. Moreover, NH_3_ can liberate hydrogen through facile catalytic decomposition or thermal decomposition [9,10]; hence, NH_3_ holds promise in addressing hydrogen storage and distribution issues [11]. Nevertheless, the conventional Haber–Bosch process suffers from high energy consumption and pollution [12,13], necessitating the urgent development of greener and more convenient synthetic methodologies. In contrast, electrocatalytic nitrogen reduction for NH_3_ synthesis reaction (NRR) features mild reaction conditions, directly utilizing H_2_O as a proton source, and harnessing the benefits of renewable energy sources such as wind and solar power [14], thereby offering potential to supplant traditional NH_3_ synthesis techniques. However, the formidable N≡N bond (941 kJ mol^−1^) and the competitive hydrogen evolution reaction (HER) significantly curtail the industrial application of NRR [15,16]. Consequently, the development of novel catalysts capable of efficiently adsorbing and activating N_2_ while concurrently suppressing HER holds immense application value.

TiO_2_ is widely used as a catalyst support in various reactions due to its economy, structural stability, and environmental friendliness [17,18]. These reactions include the oxygen evolution reaction (OER) [19], HER [20,21], nitrate reduction reaction (NO_3_RR) [22], and urea synthesis through CO_2_ + N_2_ coupling [23]. Generally, pure TiO_2_ lacks active sites for effective activation of reactants. Therefore, people often introduce active species (Cu [24], Pt [25], Fe [26], Au [27], etc.) into TiO_2_ carriers through doping to enhance the catalyst’s activity. There are also relevant reports in NRR; for example, Zhao et al. introduced phosphorus atoms into TiO_2_ nanorods to increase the catalyst’s activity by forming more oxygen vacancies. In 0.1 M LiClO_4_ electrolyte, a NH_3_ yield rate of 23.05 μmol mg_cat_^−1^ h^−1^ and a Faradaic efficiency of 12.26% were achieved at −0.3 V vs. RHE potential [28]. Yang et al. introduced Au nanoparticles into TiO_2_ nanosheets to accelerate charge transfer in the reaction, altering the local electronic structure to effectively adsorb and activate N_2_. Ultimately, at −0.4 V vs. RHE potential, a NH_3_ yield rate of 12.5 μmol mg_cat_^−1^ h^−1^ and a Faradaic efficiency of 10.2% were achieved [27]. Although these catalysts have shown decent catalytic performance, further improvement is still needed. Moreover, what is more important is that these studies are only aimed at specific elements; in other words, the selection of catalyst active sites lacks a more rational and accurate effective design.

Therefore, in our work, we first used titanate nanosheets (TiNS) as a carrier to design 12 single-atom catalyst M-TiNS (M = Fe, Co, Ni, Cu, Ru, Rh, Pd, Ag, Os, Ir, Pt, Au). Initially, we calculated the adsorption energy of N_2_ on single-atom catalyst M-TiNS, confirming that all could adsorb N_2_, with Ru-TiNS and Os-TiNS exhibiting the most negative adsorption energies. Subsequently, based on the first hydrogenation of N_2_ in NRR and HER as selection criteria, the energy barriers corresponding to each M-TiNS were calculated, confirming that Ru-TiNS and Rh-TiNS had the optimal energy barriers for the first hydrogenation of N_2_ and HER (the lower N_2_ first hydrogenation barrier and the higher HER barrier). Taking these two catalysts as examples, we calculated the Gibbs free energy of the entire reaction pathway. Among the two reaction pathways, Ru-TiNS exhibited the lowest reaction barrier, suggesting that Ru-TiNS would demonstrate superior NRR performance. Subsequently, we successfully synthesized the Ru-TiNS single-atom catalyst experimentally and demonstrated its excellent NRR performance through electrochemical experiments (NH_3_ yield rate: 15.19 μmol mg_cat_^−1^ h^−1^, Faradaic efficiency: 15.3%), which was approximately 10 times higher than that of pure TiNS. Through experiments, electronic density of states analysis, COHP calculations, and other methods, we confirmed good electron transfer between Ru sites and N_2_, thereby effectively activating N_2_ and catalyzing subsequent hydrogenation steps. Through these steps, we developed a method combining theoretical prediction with experiments to obtain excellent NRR catalysts, providing new and effective ideas for the design of novel NRR catalysts.

## 2. Materials and Methods

### 2.1. Density Fuctional Theory Calculation

All DFT calculations were conducted using the Vienna ab initio simulation package (VASP version 5.4.4) code [29,30]. The Perdew–Burke–Ernzerhof (PBE) functional within generalized gradient approximation (GGA) was employed to handle the exchange–correlation interactions [31]. The projector augmented wave (PAW) method was chosen to describe ion–electron interaction [32]. The plane-wave basis cutoff energy was set to 500 eV. The convergence thresholds of energy and force were set to 1 × 10^−5^ eV and 0.02 eV Å^−1^, respectively. A vacuum thickness of 20 Å was applied to avoid periodic interplanar interactions. The charge density difference was visualized by using the VESTA code. The Bader algorithm was employed to calculate the charge transfer and charge distribution. Additionally, for orbital-resolved chemical bonding analysis, we utilized the crystal orbital Hamilton population (COHP) method through the LOBSTER package. This method projects the PAW wave functions onto atomic-like basis functions [33,34,35,36].

The Gibbs free energy was calculated using the following equation:

G = E + E_ZPE_ − TΔS (1)

The electronic energy is denoted by E, and the zero-point energy is represented as E_ZPE_ (E_ZPE_ = 1/2Σℏv, where v is the normal mode vibrational frequency and ℏ is the reduced Planck constant). The entropy correction is designated as TΔS (with T set at 298 K). All TΔS values involved are obtained through VASPKIT using DFT-calculated frequencies, while those for gaseous molecules are sourced from the NIST-JANAF thermodynamic tables.

### 2.2. Chemicals and Material

All reagents used in the synthesis and experimental processes were not further purified. Potassium carbonate (K_2_CO_3_), potassium bicarbonate (KHCO_3_), lithium carbonate (Li_2_CO_3_), and titanium dioxide (TiO_2_, Rutile) were purchased from Alfa Aesar. Ruthenium oxide (RuO_2_) was from Acros. Tetrabutylammonium hydroxide (TBAOH) and the ammonium ion standard solution (1000 μg/mL) were purchased from Shanghai Aladdin Biochemical Technology Co., Ltd. (Shanghai, China).

### 2.3. Synthesis of TiNS and Ru-TiNS Nanosheets

TiO_2_, Li_2_CO_3_, and K_2_CO_3_ were ground in a mortar at the mole ratio in the formula (K_0.8_Ti_1.73_Li_0.27_O_4_) for 30 min. The ground powder was calcined at 800 °C for 20 h in air. And then, the sample was ground for another 30 min and calcined at 800 °C for 20 h in air again to obtain layered titanate. The second step was protonation to form layered TiO_2_ with extended interlayer distance. A total of 1 g titanate powder was dispersed in 100 mL HCl (1.5 M). The HCl needed to be replaced three times every 48 h. Then, H_0.7_Ti_1.825_O_4_ was exfoliated through dispersion in a 0.03 M tetrabutylammonium hydroxide (TBAOH) solution on a table concentrator for a duration of 10 days. The ratio of the TBAOH solution to H_0.7_Ti_1.825_O_4_ was maintained at 300 mL g^−1^. Finally, the precipitate was obtained by centrifugation, followed by thorough washing with ultrapure water to achieve a neutral pH and remove excess TBAOH. After freeze-drying for three days, fluffy titanate ultra-thin nanosheets (TiNS) were obtained.

The distinguishing factor of Ru-TiNS was the combination of several raw materials (RuO_2_, TiO_2_, Li2CO_3_, and K_2_CO_3_) in accordance with a specific molar ratio, with a molar fraction of 0.1 for the metal component.

### 2.4. Characterizations

The structure and morphology of several catalysts were characterized by X-ray diffraction (XRD, Bruker D2 diffractometer with Cu Kα radiation, Billerica, MA, USA), Scanning Electron Microscopy (SEM, Hitachi S-4800 field emission SEM, Tokyo, Japan), Transmission Electron Microscopic (TEM, Thermofisher Talos F200X with acceleration voltage of 200 kV, Waltham, MA, USA), and High Angle Annular Dark Field Scanning Transmission Electron Microscopy (HAADF-STEM, FEI Tecnai G2 F20, Waltham, MA, USA). The X-ray Photoelectron Spectroscopy (XPS) analysis was performed on a Thermo ESCALAB 250Xi electron spectrometer with 300 W Al KR radiation (Waltham, MA, USA).

### 2.5. Electrocatalytic Nitrogen Reduction Reaction (NRR) Experiment

All electrochemical characterizations were carried out using the CHI 760E (Chenhua, Shanghai, China) electrochemical workstation. A three-electrode system was employed with a platinum foil serving as the counter electrode, a saturated Ag/AgCl reference electrode, and the working electrode. A H-type electrochemical cell was utilized, separated by a Nafion 117 proton exchange membrane. Prior to use, the Nafion proton exchange membrane was boiled in a 5% H_2_O_2_ aqueous solution at 80 °C for 1 h and subjected to multiple rinses. The carbon paper used was treated before usage with a mixed solution of H_2_SO_4_ and H_2_O_2_ (1:3, vol.) for 12 h, followed by several rinses to remove surface impurities. It was then trimmed to a size of 1 × 3 cm^2^ for later use. The catalyst ink was prepared by adding 5 mg of the catalyst and 30 μL of a 5% Nafion solution to a mixture of 500 μL ethanol and 470 μL water, followed by 1 h of sonication. The working electrode was created by evenly applying 60 μL of the catalyst ink (0.3 mg of catalyst) to carbon paper (0.3 × 1 cm^2^), and allowed to air-dry naturally. The electrolyte solution was 0.1 M KHCO_3_. The potential was converted to RHE using the following equation: E (vs. RHE) = E (vs. Ag/AgCl) + 0.0591 × pH + 0.197. Before the test, N_2_ (30 mL min^−1^) was injected for 30 min, and other gas interference was discharged. Cyclic voltammetry (CV) was performed at a potential range of 0.3–0.5 V vs. RHE at a scan rate of 50 mV s^−1^ to stabilize the electrode. Subsequently, linear sweep voltammetry (LSV) was carried out at a scan rate of 10 mV s^−1^, followed by chronoamperometry (CA) testing.

The generated NH_3_ was quantitatively analyzed using the indophenol blue method [37]. A total of 2 mL post-reaction electrolyte was taken from the cathode chamber. Then, 1 mL of the diluted electrolyte was mixed with 2 mL of 1.0 M NaOH solution (including 5 wt% salicylic acid and 5 wt% sodium citrate), followed by the addition of 1 mL of 0.05 M NaClO solution and 0.2 mL of 1 wt% sodium nitroprusside solution. After thorough mixing, the mixture was allowed to stand at room temperature for 2 h. UV-Vis absorption spectroscopy data were collected at a wavelength of 655 nm. Subsequently, the concentration of NH_3_ was calculated using a standard curve.

The NH_3_ yield rate (μg h^−1^ mg_cat_^−1^) is calculated using the following formula:

$${Yield\;rate}_{NH3}=(C_{NH3}\times V)/(t\times m_{cat})$$

The Faradaic Efficiency (FE) for NH_3_ is calculated using the following formula:

$${FE}_{NH3}=\frac{{3\times C}_{NH3}\times V\times F\times 10^{-6}}{17\times Q}\times 100\%$$

In this equation, C_NH3_ represents the concentration of NH_3_ detected in the catholyte (μg mL^−1^); V is the volume of the catholyte in the cathode compartment (30 mL); t stands for the reaction time (1 h); m_cat_ indicates the mass of the loaded catalyst (mg); F is Faraday’s constant (96,485 C mol^−1^); and Q refers to the total charge transferred during the reaction (C).

## 3. Results and Discussion

### 3.1. Theoretical Screening

**Figure 1:**
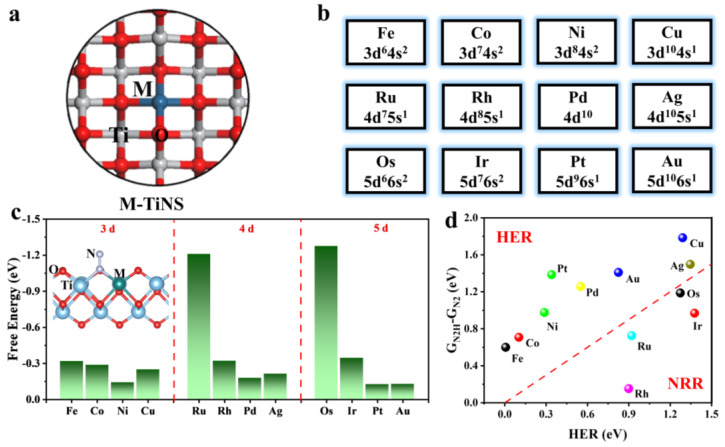
(**a**) Presents the top view of the M-TiNS model, employed for introducing the M element into TiNS, as shown in (**b**). (**c**) Free energy of N_2_ adsorption on M-TiNS. The illustration depicts the adsorption configuration of N_2_. (**d**) The selectivity of NRR over HER is delineated by the changes in Gibbs free energy (ΔG) for the initial hydrogenation of N_2_ (N_2_ + H^+^ + e^−^ → NNH) and hydrogen recombination (H^+^ + e^−^ → 1/2H_2_) on M-TiNS, displayed along the horizontal and vertical axes, respectively. The dashed line in the figure represents the equality of ΔG for the two processes.

All models of M-TiNS catalysts were created by substituting M atoms for five-coordinate Ti atoms in the lattice, which has been confirmed in previous studies [38]. The lattice parameters are a = 14.99 Å and b = 12.11 Å, with a vacuum layer of 20 Å added in the c direction to prevent periodic interlayer interactions (Figure 1a,b). Group VIII and IB transition metals were selected as the active centers to construct single-atom catalysts, and the corresponding structures are shown in Figure S1.

The prerequisite for the nitrogen reduction reaction (NRR) is the efficient adsorption and activation of N_2_ at the active site. Figure 1c shows the adsorption energy of N_2_ on M-TiNS, with the corresponding adsorption structures depicted in Figure S2. The results indicate that all catalysts adopt an end-on adsorption model for N_2_, and their adsorption energies are negative values, suggesting that the process is exothermic and stable. Notably, the Ru and Os-TiNS catalysts exhibit adsorption energies of −1.21 eV and −1.28 eV, respectively. The adsorption energies for other catalysts are around −0.3 eV. This suggests that N_2_ adsorption is most stable on these two catalysts. Concurrently, we computed the post-adsorption N-N bond length; for all catalysts, the N-N bond is elongated following N_2_ adsorption, exceeding the ideal N_2_ molecule’s 1.10 Å (Figure S3), which signifies effective activation of the N_2_ molecule. Subsequently, the average bond lengths for M-N and Ti-N were tallied (Figure S4), oscillating between 2.1 and 2.8 Å, which is less than the distance characteristic of van der Waals interactions, confirming the likelihood of the N_2_ adsorption being chemisorptive in nature. Bader charge analysis revealed directional electron transfer from the M atoms to the N_2_ molecule (Figure S5). Furthermore, the trend in the quantity of charge transfer differed from that of the bond length (or adsorption energy), which could be attributed to the influence of spin electrons.

During the N_2_ electrocatalysis process, the HER (hydrogen evolution reaction) is unavoidable; it competes with the NRR for protons and electrons in the electrolyte, leading to a decrease in NRR activity. The free energy of *H is significantly affected by the applied electric field, primarily due to its involvement in proton and electron transfer. In contrast, the adsorption process of N_2_ lacks proton and electron transfer, and its free energy is unaffected by the electric potential. Therefore, we compared the free energies of N_2_ hydrogenation to NNH and H recombination to evaluate the selectivity of the catalysts (Figures S6 and S7). As shown in Figure 1d, most catalysts exhibit a catalytic activity in HER that significantly affects the Faradaic efficiency of NRR, promoting hydrogen production via HER. However, Rh, Ru, Os, and Ir-TiNS catalysts mainly promote NRR. Nonetheless, Os and Ir-TiNS catalysts with hydrogenation barriers greater than 0.8 eV were excluded. Consequently, Rh and Ru-TiNS catalysts emerge as promising candidates for effectively suppressing HER in ammonia synthesis.

**Figure 2:**
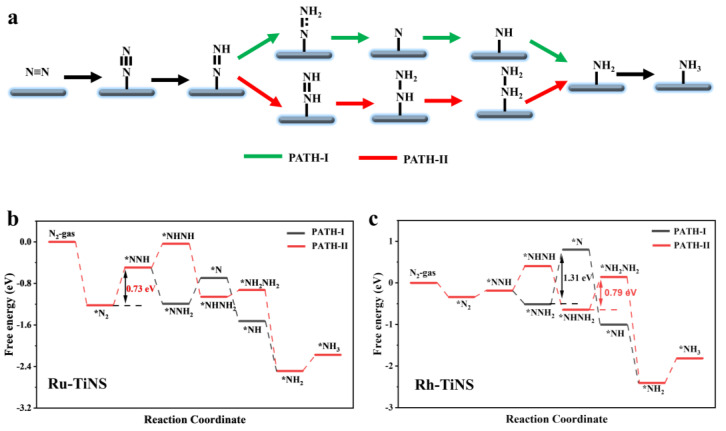
(**a**) Illustrated schematically are the distal and alternating pathways for NRR. (**b**,**c**) Gibbs free energy profiles for the electroreduction of N_2_ on Ru-TiNS and Rh-TiNS.

As is widely recognized, there are two probable reaction mechanisms for NRR: the distal hydrogenation mechanism (PATH-I) [39] and the alternating hydrogenation mechanism (PATH-II) [40] (Figure 2a). In the distal pathway, the proton–electron pair initially attacks the distal nitrogen atom consecutively to release one molecule of NH_3_, followed by an attack on the second nitrogen atom to release a second NH_3_ molecule, thus completing the reaction. Conversely, in the alternating pathway, the proton–electron pair alternately attacks the two nitrogen atoms to simultaneously form two NH_3_ molecules.

Figure 2b,c show the Gibbs free energy diagrams for the NRR on Ru and Rh-TiNS catalysts via the distal and alternating pathways. The configurations of the relevant reaction intermediates are presented in Figures S8 and S9. Initially, the adsorption of N_2_ on the catalysts is an end-on process and exothermic. Subsequently, the *N_2_ is attacked by a proton–electron pair (H^+^/e^−^) to form *NNH, completing the first hydrogenation step. The Gibbs free energy changes (ΔG) for Ru and Rh-TiNS are 0.73 eV and 0.15 eV, respectively. For the Ru-TiNS catalyst, this represents the rate-determining step (RDS) for both PATH-I and PATH-II. Thereafter, *NNH undergoes further attack by H^+^/e^−^, with two possible outcomes: formation of either the *NHNH or *NNH_2_ intermediate. The formation of *NHNH on Ru and Rh-TiNS requires the absorption of 0.46 eV and 0.59 eV of energy, respectively, while the formation of *NNH_2_ is exothermic. Thus, both catalysts complete the reaction via PATH-I. Following this, *NNH_2_ is attacked by H^+^/e^−^ to form the *N intermediate and release one molecule of NH_3_. This step is endothermic for both catalysts, with ΔG values of 0.50 and 1.31 eV, respectively. For the Rh-TiNS catalyst, this is the RDS for the entire reaction process (path-I). Furthermore, in the case of Rh-TiNS in path-II, the RDS barrier is 0.79 eV (NHNH_2_ → NH_2_NH_2_). Given that the RDS for Rh-TiNS is substantially higher than that for Ru-TiNS, only the Ru-TiNS catalyst is discussed henceforth. After the release of the first NH_3_ molecule, *N undergoes a three-step protonation process, i.e., *N → *NH → *NH_2_ → *NH_3_, to produce the second NH_3_ molecule. The first two steps are exothermic, while the third step requires the absorption of 0.31 eV. Subsequently, the Bader charges of the intermediates during the reaction process were calculated (Figure S10).

### 3.2. Characterization of Model Catalyst (Ru-TiNS)

Due to the implications from theoretical calculations suggesting the potentially excellent NRR performance of Ru-TiNS, we embarked on the experimental synthesis of Ru-TiNS, aiming to validate the conclusions drawn from theoretical computations.

**Figure 3:**
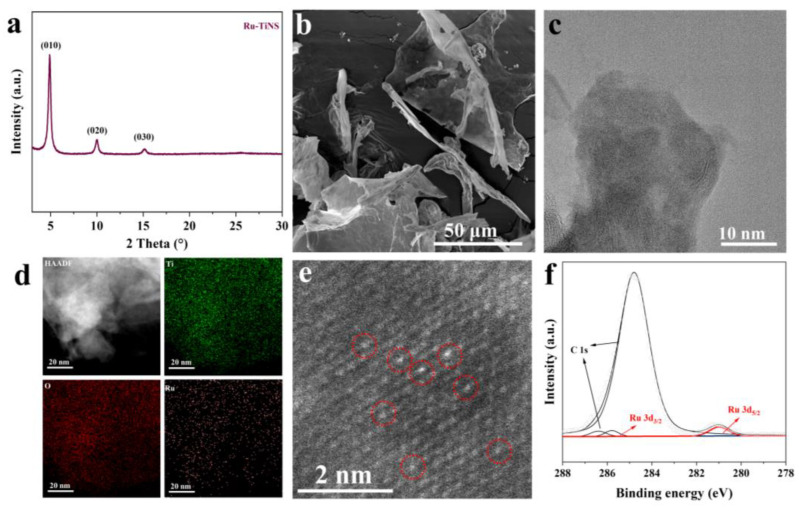
Structural characterization of the synthesized model catalyst Ru-TiNS. XRD pattern of Ru-TiNS (**a**), SEM image (**b**), and HRTEM image (**c**). (**d**) EDS element mappings of the Ru-TiNS catalyst. (**e**) Aberration-corrected HAADF-STEM image, where the Ru single atoms are highlighted by red circles. (**f**) High-resolution XPS spectrum of Ru in Ru-TiNS.

**Figure 4:**
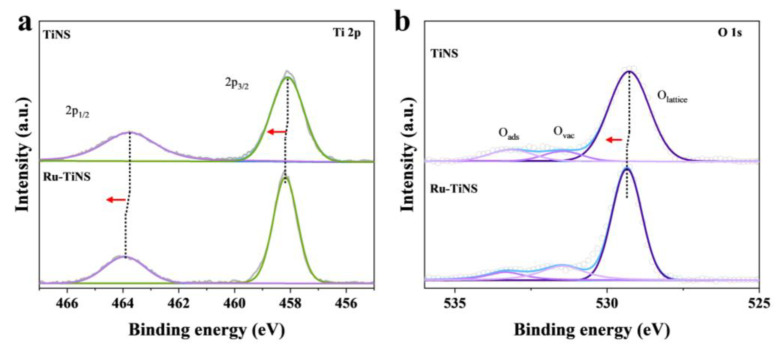
(**a**) High-resolution XPS spectrum of Ti in Ru-TiNS and TiNS. (**b**) High-resolution XPS spectrum of O in Ru-TiNS and TiNS.

Following our previous methodology [38,41], Ru-doped TiNS catalyst was synthesized using a combination of solid-state grinding, calcination, ion exchange, and soft-template stripping techniques. Inductively coupled plasma optical emission spectrometry (ICP-OES) characterization confirmed the Ru loading to be 3.6 wt%. Initially, X-ray diffraction (XRD) patterns (Figure 3a) revealed characteristic off-plane diffraction peaks of typical 0k0 (k = 1, 2, 3) stacked layered structures in Ru-TiNS [23,42,43,44]. Further visual examination via scanning electron microscopy (SEM) (Figure 3b) displayed the distinct layered structure of Ru-TiNS. This result is consistent with the SEM images of pure TiNS (Figure S11), and combined with their similar BET surface areas (Figure S12), it suggests that the introduction of Ru does not lead to morphological differences. Moreover, a high-resolution transmission electron microscopy (HRTEM) image illustrated its ultrathin layered structure (Figure 3c). Through high-angle annular dark-field scanning TEM (HAADF-STEM) imaging, no aggregated Ru species were detected, consistent with the corresponding elemental mapping results. (Figure 3d). To gain deeper insights into the state of Ru, aberration-corrected TEM characterization was performed. As depicted in Figure 3e, Ru atoms were uniformly distributed within the TiNS lattice through replacing Ti atoms, consistent with the theoretical model constructed. X-ray photoelectron spectroscopy (XPS) analysis revealed characteristic peaks attributed to Ru^3+^ species at a binding energy of 281.1 and 285.7 eV, indicating the Ru species were in the +3 valance state (Figure 3f) [45]. This result is consistent with the Ru 3p XPS (Figure S13) [46]. It can be observed that compared to the original RuO_2_ with a +4 valence state, Ru was partially reduced. Furthermore, through XPS spectra of Ti and O, it can be observed that, compared to pure TiNS support, characteristic peaks of Ti and O have shifted towards higher binding energies, indicating partial electron transfer from the TiNS support to Ru [47,48,49,50,51,52], which is consistent with the change in the valence state of Ru. (Figure 4a,b). This phenomenon indicated a strong interaction between Ru and the TiNS support. Furthermore, from the XPS spectrum of O, it can be observed that the introduction of Ru also leads to an increase in oxygen vacancies (O_vac_), suggesting that Ru replaces Ti in the TiO_6_ octahedral sites, forming unsaturated coordination structures of RuO_x_ (x < 6), consistent with the structure predicted by our theoretical modeling. Drawing from these characterization findings, we ascertain the successful synthesis of single-atom Ru-doped ultrathin layered TiNS catalyst.

### 3.3. NRR Performance Test of Ru-TiNS

To evaluate the performance of Ru-TiNS for electrocatalytic N₂ reduction to produce NH_3_, we employed a H-type electrolysis cell and utilized carbon paper (CP) loaded with Ru-TiNS as the working electrode, forming a three-electrode system alongside a saturated Ag/AgCl reference electrode and a platinum foil electrode serving as the counter electrode. A 0.1 M KHCO₃ solution saturated with N₂ was used as the electrolyte, with continuous N₂ flow (30 mL min^−1^) throughout the testing period. Prior to testing, cyclic voltammetry (CV) tests were conducted in the non-Faradaic region (0.3–0.4 V vs. RHE) until stability was achieved to activate the electrode.

**Figure 5:**
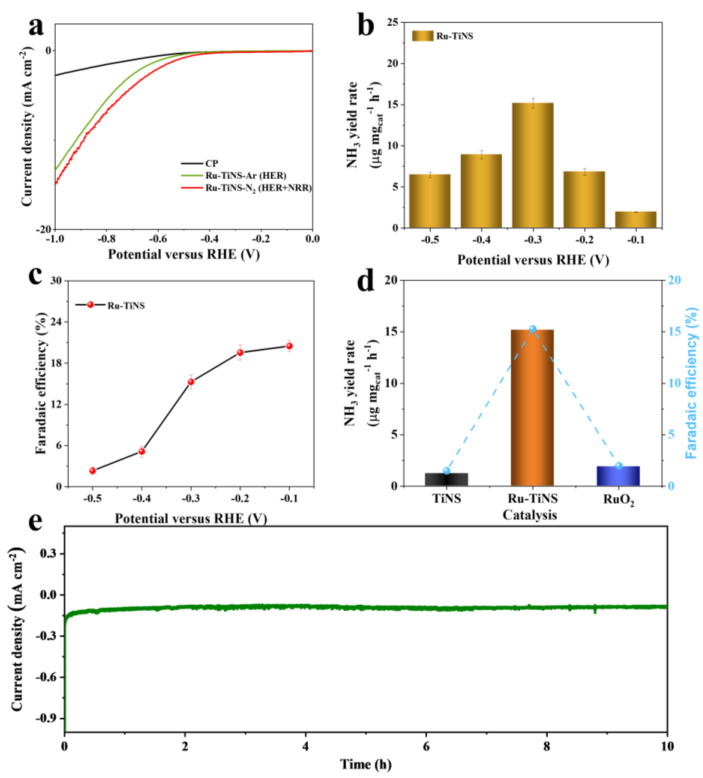
Electrocatalytic NRR performance of TiNS and Ru-TiNS. (**a**) Linear sweep voltammetry curves of CP in electrolyte saturated with nitrogen, Ru-TiNS in electrolyte saturated with argon, and electrolyte saturated with nitrogen. (**b**) NH_3_ yield of Ru-TiNS at different potentials (**b**) and Faradaic efficiency (**c**). (**d**) Comparison of catalytic performance between TiNS, RuO_2_, and Ru-TiNS at −0.3V vs. RHE. (**e**) Stability test of Ru-TiNS at −0.3 V vs. RHE for 10 h.

Firstly, linear sweep voltammetry (LSV) tests were performed in the potential range of −1 to 0 V vs. RHE to preliminarily assess the catalytic activity of Ru-TiNS under different atmospheres. As shown in Figure 5a, under an argon atmosphere, the reaction occurring on Ru-TiNS is HER. Compared to the current density curve obtained after Ar saturation of the electrolyte, Ru-TiNS exhibited higher current density under N₂ atmosphere. Moreover, compared to pure CP, Ru-TiNS showed a significant increase in current density, indicating its promising NRR catalytic activity. To quantitatively analyze the catalytic performance of Ru-TiNS, we conducted chronoamperometry (CA) tests at different potentials for 1 h. As shown in Figure S14, with increasingly negative potentials, the current density also increased, suggesting more vigorous reactions occurring on the electrode surface. After 1 h of reaction, quantitative analysis of NH_3_ concentration in the electrolyte was performed using the indophenol blue method combined with standard curves (Figures S15 and S16), as shown in Figure 5b,c. It can be observed that the NH_3_ yield corresponding to Ru-TiNS exhibited a volcano-shaped relationship with potential. The highest NH_3_ yield (15.19 μmol mgcat^−1^ h^−1^) and satisfactory Faradaic efficiency (15.3%) were achieved at −0.3 V vs. RHE. The Watt and Chrisp method was used to detect potential byproduct N_2_H_4_ [53], and the results indicated the absence of N_2_H_4_ (Figure S17). Additionally, we calculated the FE of H_2_ (Figure S18), which increased as the potential became more negative. At higher potentials, N₂ activation was ineffective due to insufficient driving force, while at lower potentials, competition from hydrogen evolution reactions led to a decrease in NRR performance, as inferred from the reduced Faradaic efficiency and NH_3_ yield after −0.3 V vs. RHE. To verify the source of the product, various experiments were designed (Figure S19). At −0.3 V vs. RHE, when using pure CP as the working electrode, NH_3_ was not detected in the product; however, NH_3_ was only detected when N₂ was introduced during the reaction with Ru-TiNS as the working electrode, and no NH_3_ was detected after one hour of electrolysis at open circuit potential (OCP). Combined with the absence of external nitrogen in the catalyst, it can be concluded that NH_3_ in the product originates from the introduced N₂. Furthermore, to determine the active center, CA tests were conducted on pure TiNS support, as shown in Figure 5d. It can be observed that at the optimal potential of −0.3 V vs. RHE, TiNS exhibited a NH_3_ yield of only 1.23 μmol mgcat^−1^ h^−1^, with a corresponding FE of 1.5%. In comparison, the NH_3_ yield and FE of Ru-TiNS were increased by ~10 times, indicating that the introduction of Ru effectively enhances the catalyst’s activity. By comparing the catalytic performance with the pure TiNS support, we demonstrate that the ultrathin TiNS support lacks the ability to catalyze NRR. This further supports that the NRR active sites on Ru-TiNS are attributed to Ru sites. Additionally, we further tested the activity of RuO_2_ and found that RuO_2_ has almost no catalytic activity. By comparing it with Ru-TiNS, we demonstrate that the unique chemical structure of Ru sites in the synthesized Ru-TiNS contributes to its excellent catalytic activity in the NRR process. In the 10 h long-term stability test (Figure 5e), there was no significant decay in the corresponding current density of Ru-TiNS, demonstrating the excellent stability of the Ru-TiNS catalyst.

Subsequently, to evaluate the electrochemical active area of Ru-TiNS, CV tests were conducted in the non-Faradaic region, obtaining the CV curves of Ru-TiNS catalyst and pure TiNS support at different scan rates (Figure S20). By linear fitting of the difference in current density and scan rate in the CV curves, the slope of the straight line obtained represents the electrochemical double-layer capacitance (C_dl_) [54]. The C_dl_ values of Ru-TiNS and pure TiNS support were 9.94 mF cm^−2^ and 0.66 mF cm^−2^, respectively. A larger C_dl_ value indicates that Ru-TiNS exposes more catalytic active sites, further confirming the role of Ru as the active site.

Furthermore, EIS impedance tests were conducted on Ru-TiNS and TiNS separately, as shown in the Nyquist plot in Figure S21. Both exhibited semicircles in the high-frequency range of the impedance spectrum. The radius of the semicircle in the plot corresponding to Ru-TiNS was much smaller than that of TiNS, indicating more intense electron transfer between Ru and N₂, consistent with the theoretical calculations mentioned below [55].

### 3.4. Interaction between Ru-TiNS and Reactants

**Figure 6:**
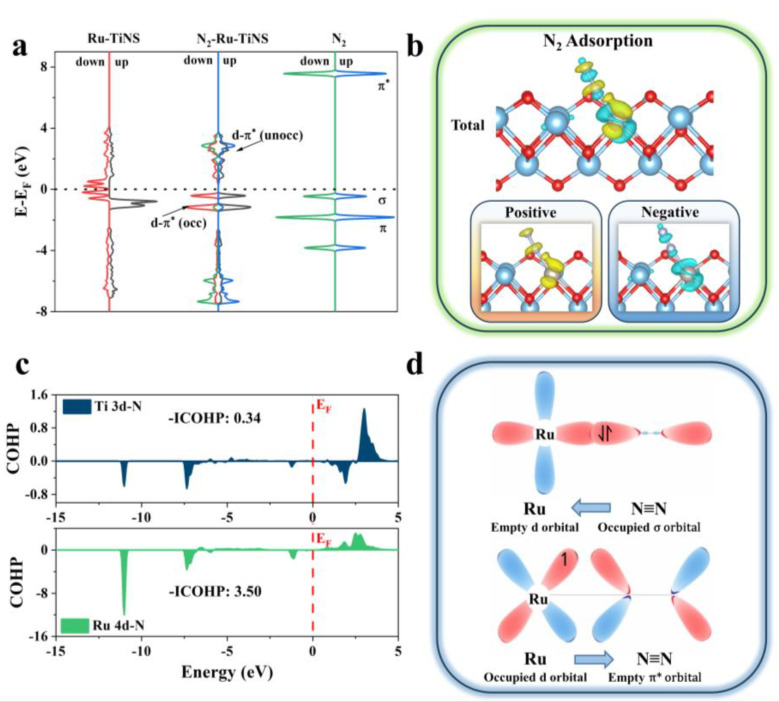
(**a**) The density of states (DOS) diagrams for free N_2_, Ru-TiNS, and N_2_ adsorbed on Ru-TiNS. (**b**) The charge density distribution after N_2_ adsorption on Ru-TiNS. Blue and yellow represent charge depletion and charge accumulation, respectively. (**c**) The crystal orbital Hamilton populations (COHP) for *N_2_ adsorbed on Ru-TiNS. (**d**) A schematic illustration of the interaction between N_2_ and Ru-TiNS.

In order to further understand the interaction between N_2_ and Ru-TiNS, the density of states (DOS) was calculated initially. As shown in Figure 6a, Ru-TiNS exhibits significant spin polarization, with the up-spin orbitals substantially higher than the down-spin orbitals, possessing an absolute spin of 2 μ_B_. Upon the adsorption of N_2_, the spin of Ru-TiNS vanishes, which evidences the substantial impact of spin electrons in the N_2_ activation process (Figures S22 and S23). What is more, the unoccupied d orbitals of Ru-TiNS accept electrons from the σ and π orbitals of N_2_, which are in proximity to the Fermi level. This is likely due to the principal involvement of Ru-up orbitals, leading to their reduction and even appearance below the Fermi level, thus forming a bonded state and enhancing N_2_ adsorption. Furthermore, the occupied d orbital electrons of Ru-TiNS are returned to the π* orbitals of N_2_, leading to partial occupancy and expansion below the Fermi level of N_2_. In other words, the activation of N_2_ follows an “acceptance-donation” mechanism [56]. Simultaneously, Figure 6d presents a schematic of this process. As depicted in Figure 6b, the charge density difference further verifies this mechanism, with the feedback mechanism playing a dominant role, resulting in a net charge transfer of 0.40e from Ru-TiNS to N_2_.

Moreover, COHP reveals the strength of the d-π interaction, with -ICOHP given quantitatively. For the adsorbed configuration of N_2_, the -ICOHP values for Ti 3d-N and Ru 4d-N are 0.34 and 3.50, respectively (Figure 6c). The disparity greater than an order of magnitude confirms the decisive role of Ru incorporation in the reactivity of N_2_.

## 4. Conclusions

In summary, to obtain a superior NRR catalyst, we conducted theoretical screening of 12 M-TiNS catalysts and confirmed that Ru-TiNS had the optimal NRR rate-determining step energy barrier, as well as a higher HER energy barrier, implying that Ru-TiNS exhibits excellent NRR catalytic performance and can suppress the occurrence of the HER reaction. Subsequently, at the experimental level, we successfully synthesized the Ru-TiNS single-atom catalyst and found through electrochemical testing that Ru-TiNS exhibited outstanding activity at −0.3 V vs. RHE: NH_3_ yield rate, reaching 15.19 μmol mg_cat_^−1^ h^−1^ and a Faradaic efficiency (FE) of 15.3%. This performance is approximately 10 times higher than that of pure TiNS. Through electronic density of states analysis and COHP analysis, we confirmed good electron transfer between Ru and N_2_, enabling N_2_ activation and hydrogenation. Through these findings, we have successfully developed a method aimed at providing unique insights and approaches for the rational design and synthesis of efficient NRR catalysts.

## Data Availability

Data are contained within the article.

## Supplementary Materials

The following supporting information can be downloaded at: https://www.mdpi.com/article/10.3390/ma17102239/s1, Figure S1: The adsorption configurations of M-TiNS; Figure S2: The adsorption configurations of N_2_ on M-TiNS; Figure S3: N-N bond length after N_2_ adsorption; Figure S4: M-N bond length after N_2_ adsorption; Figure S5: Charge changes after N_2_ adsorption; Figure S6: The adsorption configurations of *H on M-TiNS; Figure S7: The adsorption configurations of *NNH on M-TiNS; Figure S8: Configuration of intermediates in N_2_ reduction reaction on Ru-TiNS; Figure S9: Configuration of intermediates in N_2_ reduction reaction on Rh-TiNS; Figure S10: Bader charges of intermediates along the reaction coordinates for in N_2_ reduction reaction on Ru-TiNS; Figure S11: SEM image of pure TiNS support; Figure S12: Nitrogen adsorption–desorption isotherms and their corresponding surface area of TiNS (a)and Ru-TiNS (b); Figure S13: High-resolution Ru 3p XPS spectrum of Ru in Ru-TiNS; Figure S14: The i–t curves obtained from chronoamperometry tests at different potentials; Figure S15: The UV-visible absorption curves corresponding to the coloration of indophenol blue after reaction with NH_4_^+^ of different concentrations; Figure S16: The linear fitting curves of absorbance at 655 nm after coloration by the indophenol blue method for NH_4_^+^ of different concentrations; Figure S17: UV-Vis absorbance curves of Ru-TiNS before and after electrolysis at −0.3 V vs. RHE; Figure S18. FE_H2_ of Ru-TiNS at different potentials; Figure S19: NH_3_ yield rates under different conditions including pure carbon paper (−0.3 V vs. RHE), open circuit potential, only Ar bubbling (−0.3 V vs. RHE), and N_2_ bubbling (−0.3 V vs. RHE); Figure S20: CV curves obtained for pure TiNS (a) and Ru-TiNS (b) at different scan rates, along with the corresponding calculated double-layer capacitances (c); Figure S21: Electrochemical impedance spectroscopy (EIS) of Ru-TiNS and TiNS; Figure S22: The Spin charge density of (a) Ru-TiNS and (b) N_2_ adsorption on Ru-TiNS; Figure S23: The projected density of (a) Free N_2_, (b) Ru 4d in Ru-TiNS, and (c,d) N_2_ molecule adsorbed on Ru-TiNS.
