# Supplementary material for: Efficient Electrocatalytic Ammonia Synthesis via Theoretical Screening of Titanate Nanosheet-Supported Single-Atom Catalysts
Source: Materials (Basel). 2024 May 9;17(10):2239. doi: 10.3390/ma17102239 (PMC11123175; doi:10.3390/ma17102239)
Supplement: Supplementary file 1 [file materials-17-02239-s001.zip › materials-2964370-supplementary.pdf]

materials

*Supplementary materials*

# Efficient Electrocatalytic Ammonia Synthesis via Theoretical Screening of Titanate Nanosheet-Supported Single-Atom Catalysts

**Kaiheng Zhao [1], Jingnan Wang [1], Yongan Yang [1,*] and Xi Wang [2,3,*]**

[1] Institute of Molecular Plus, School of Chemical Engineering and technology, Tianjin University, Tianjin 300072, China
[2] Key Laboratory of Luminescence and Optical Information, Ministry of Education, School of Physical Science and Engineering, Beijing Jiaotong University, Beijing 100044, China
[3] Tangshan Research Institute of Beijing Jiaotong University, Tangshan 063000, China
* Correspondence: xiwang@bjtu.edu.cn (X.W.); revned_yang@tju.edu.cn (Y.Y.)

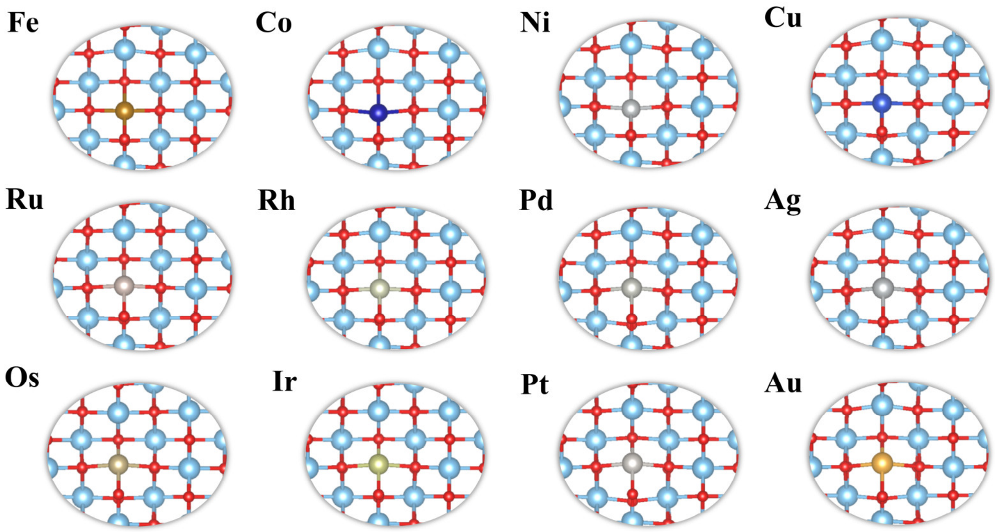


**Figure S1.** The adsorption configurations of M-TiNS.

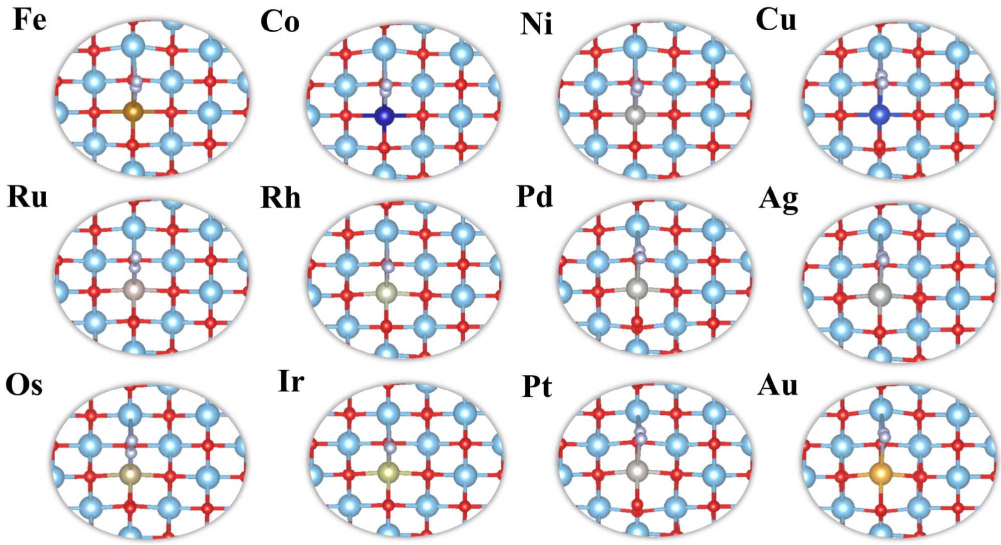


**Figure S2.** The adsorption configurations of $N_2$ on M-TiNS.

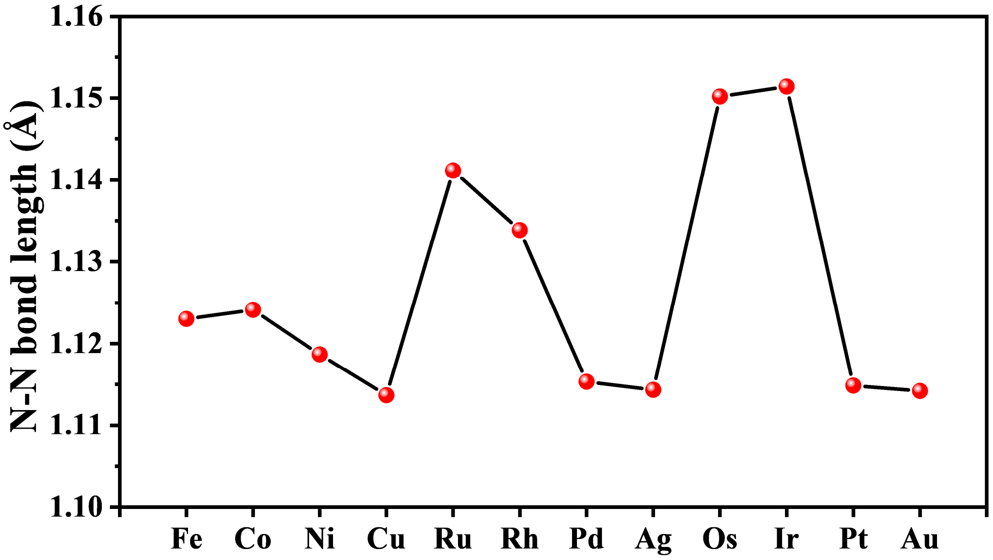


**Figure S3.** N-N bond length after $N_2$ adsorption.

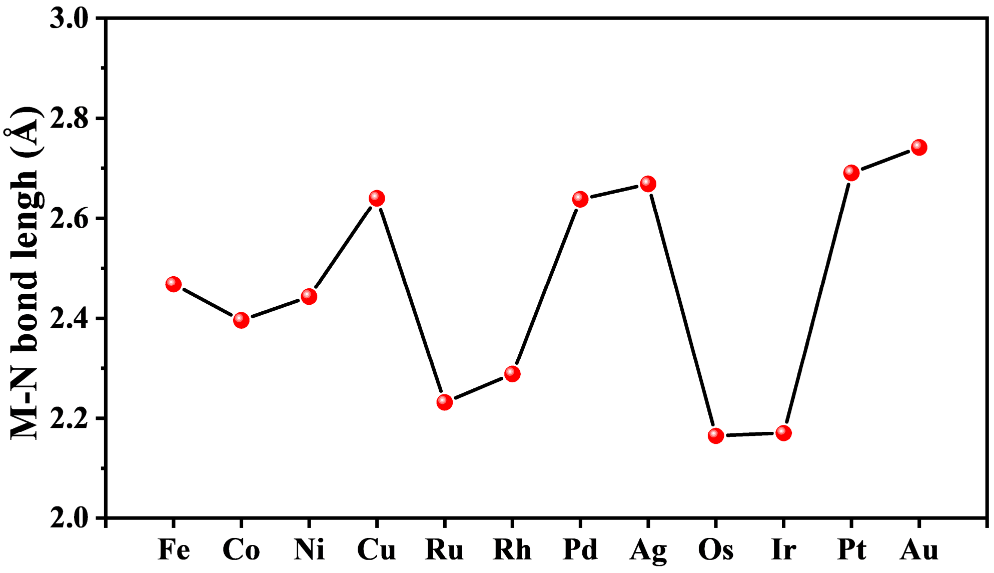


**Figure S4.** M-N bond length after $N_2$ adsorption.

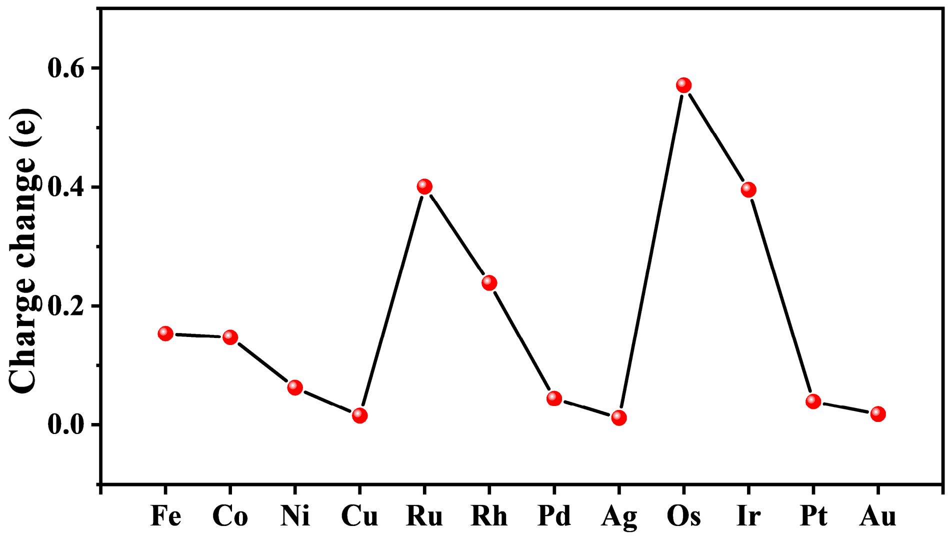


**Figure S5.** Charge changes after $N_2$ adsorption.

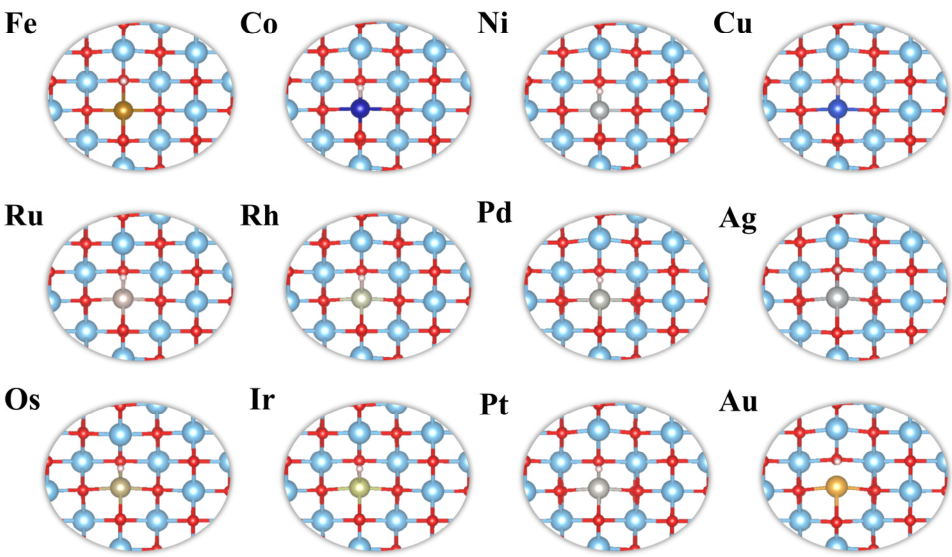


**Figure S6.** The adsorption configurations of *H on M-TiNS.

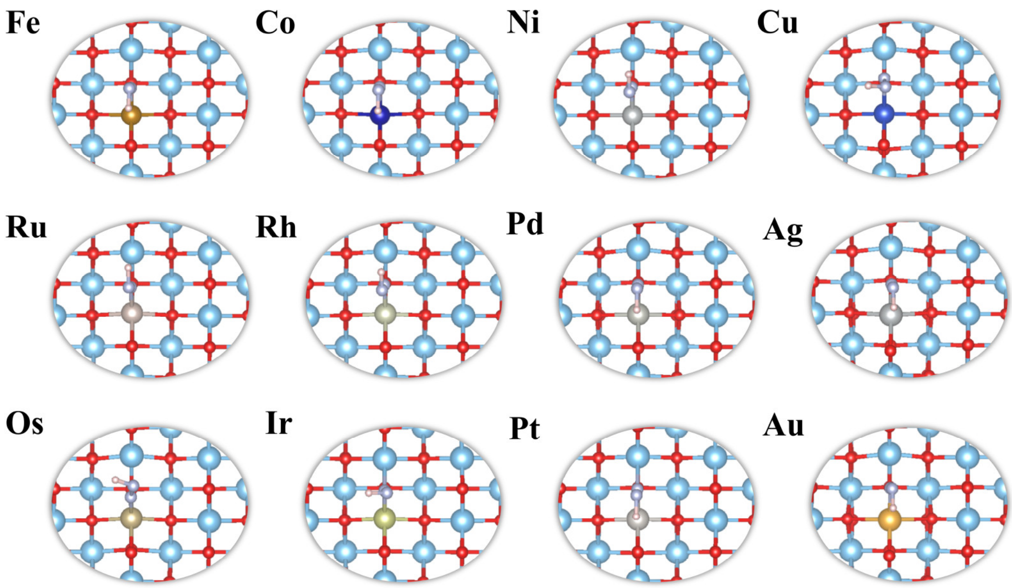


**Figure S7.** The adsorption configurations of *NNH on M-TiNS.

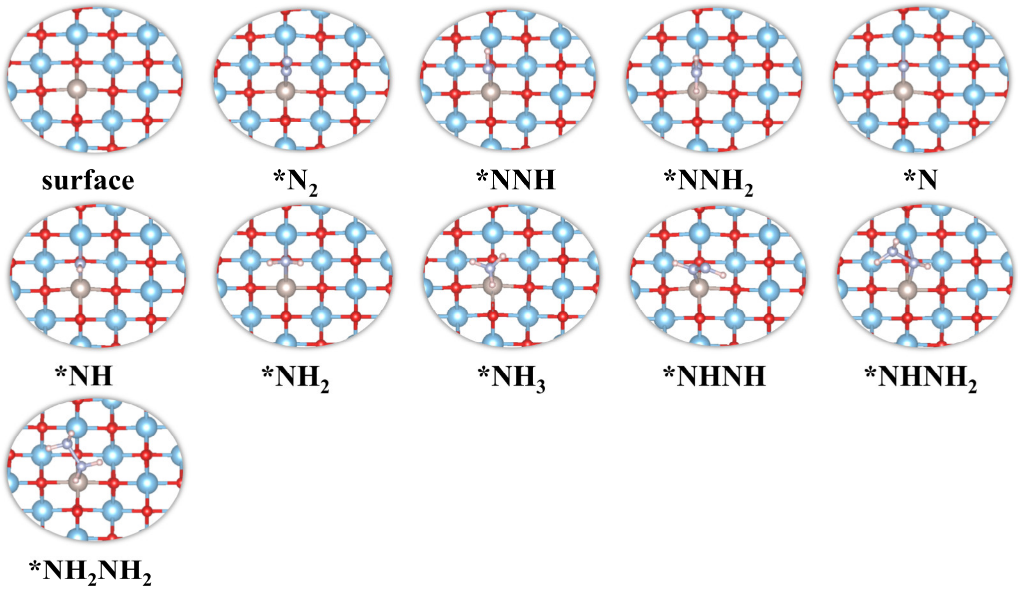


**Figure S8.** Configuration of intermediates in $N_2$ reduction reaction on Ru-TiNS.

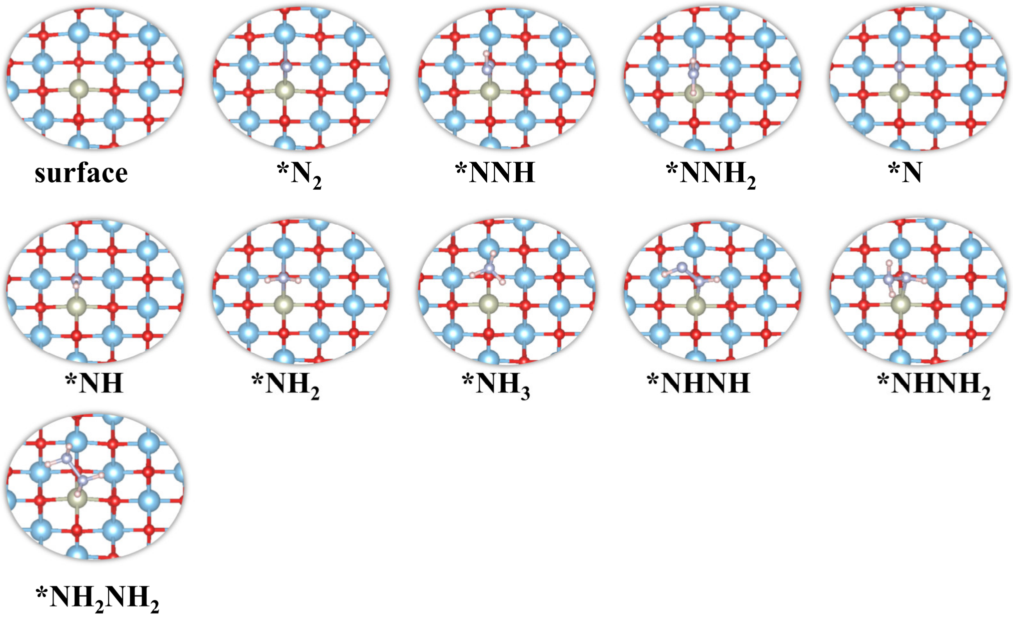


**Figure S9.** Configuration of intermediates in $N_2$ reduction reaction on Rh-TiNS.

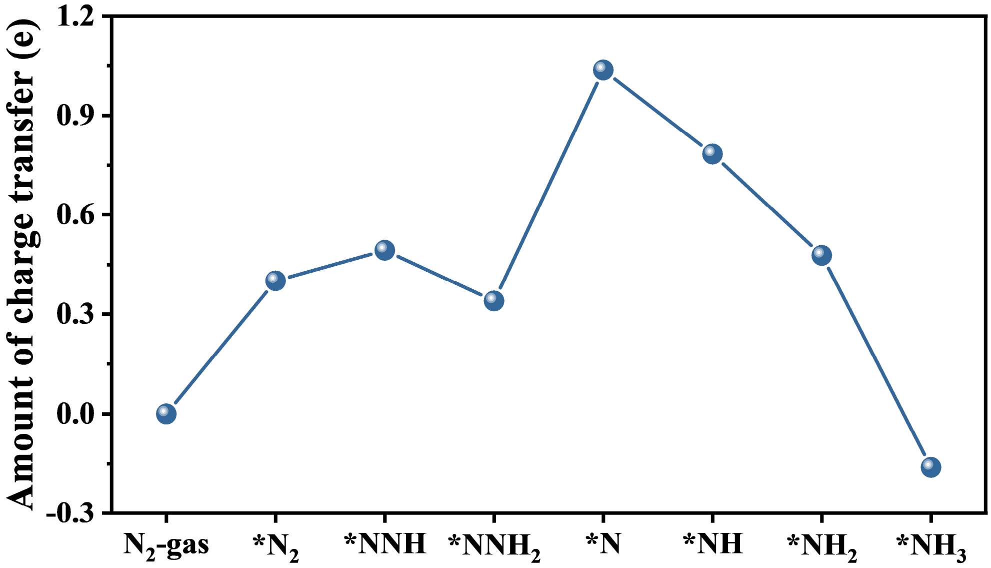


**Figure S10.** Bader charges of intermediates along the reaction coordinates for in $N_2$ reduction reaction on Ru-TiNS.

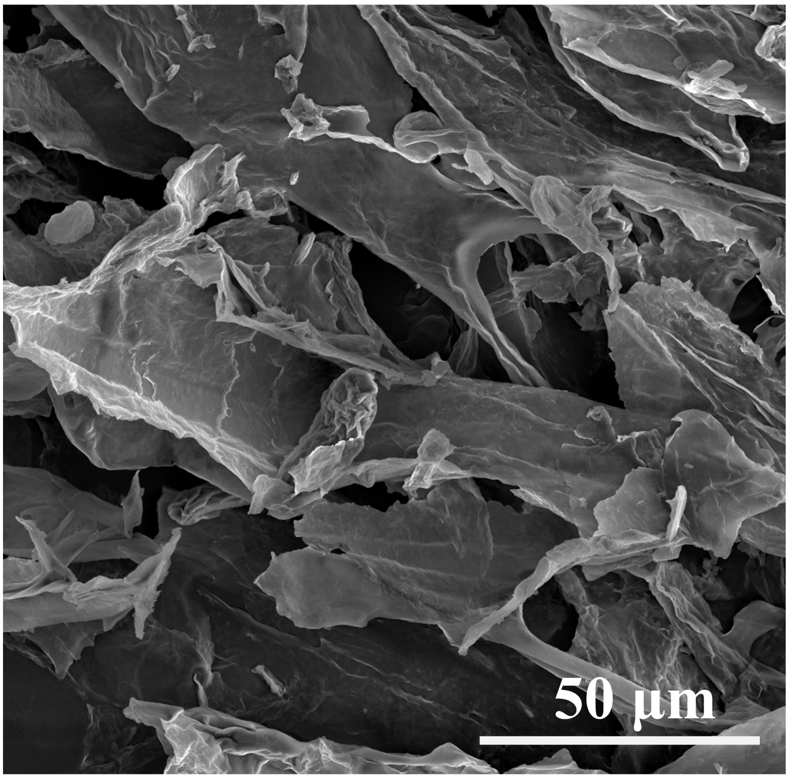


**Figure S11.** SEM image of pure TiNS support.

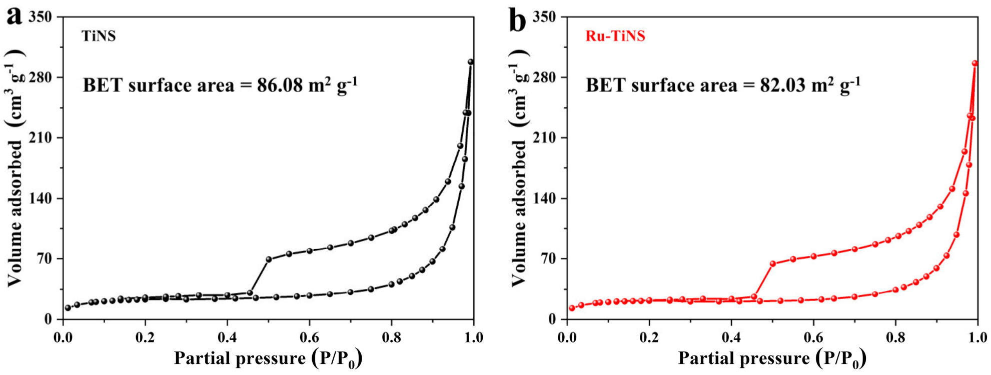


**Figure S12.** Nitrogen adsorption–desorption isotherms and their corresponding surface area of TiNS (a) and Ru-TiNS (b).

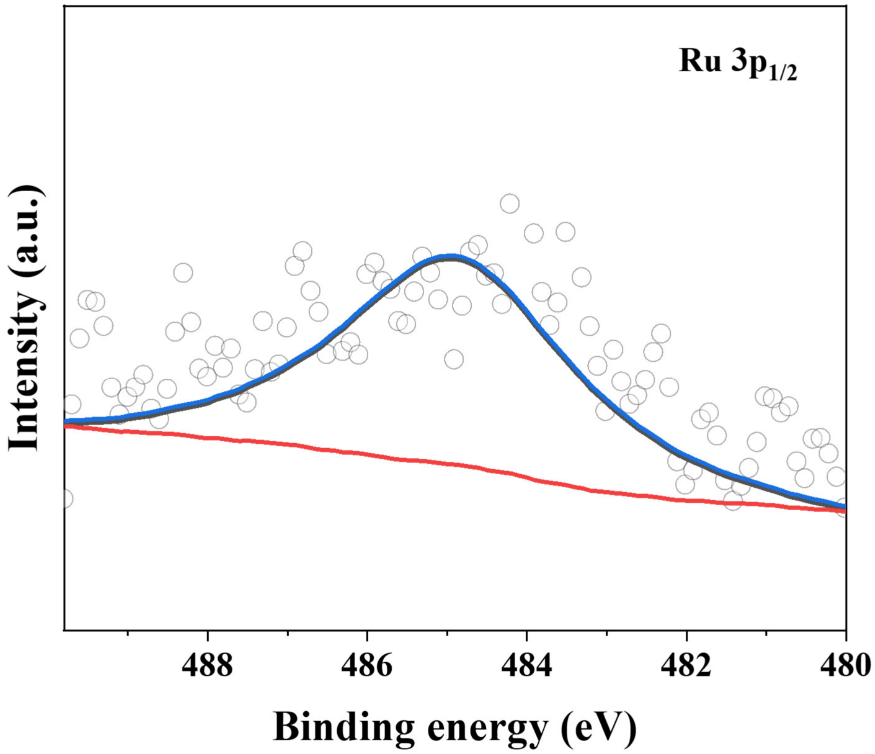


**Figure S13.** High-resolution Ru 3p XPS spectrum of Ru in Ru-TiNS.

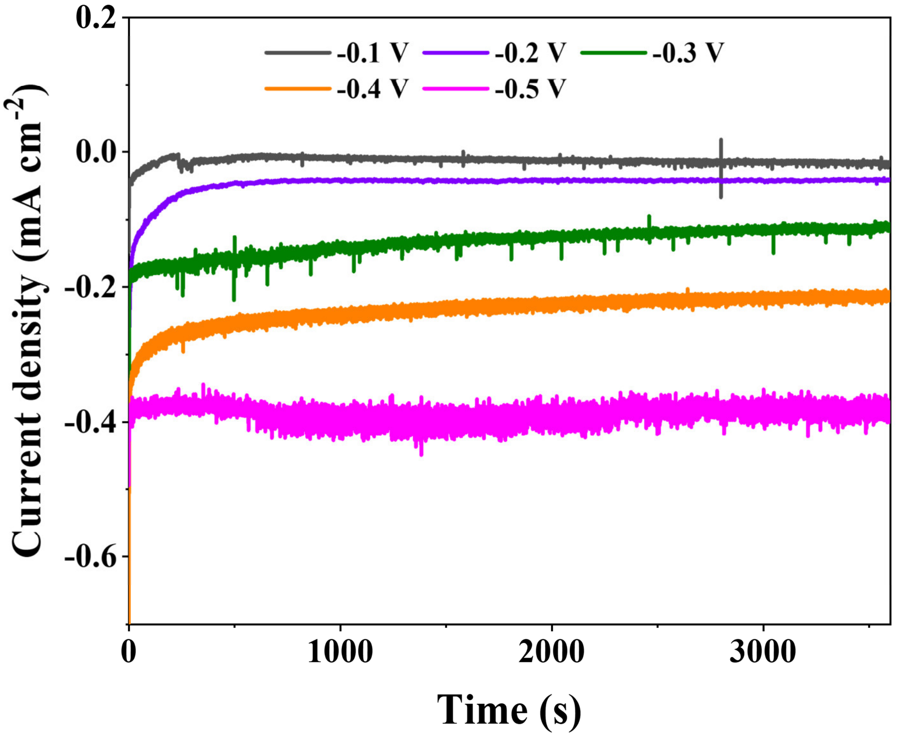


**Figure S14.** The i-t curves obtained from chronoamperometry tests at different potentials.

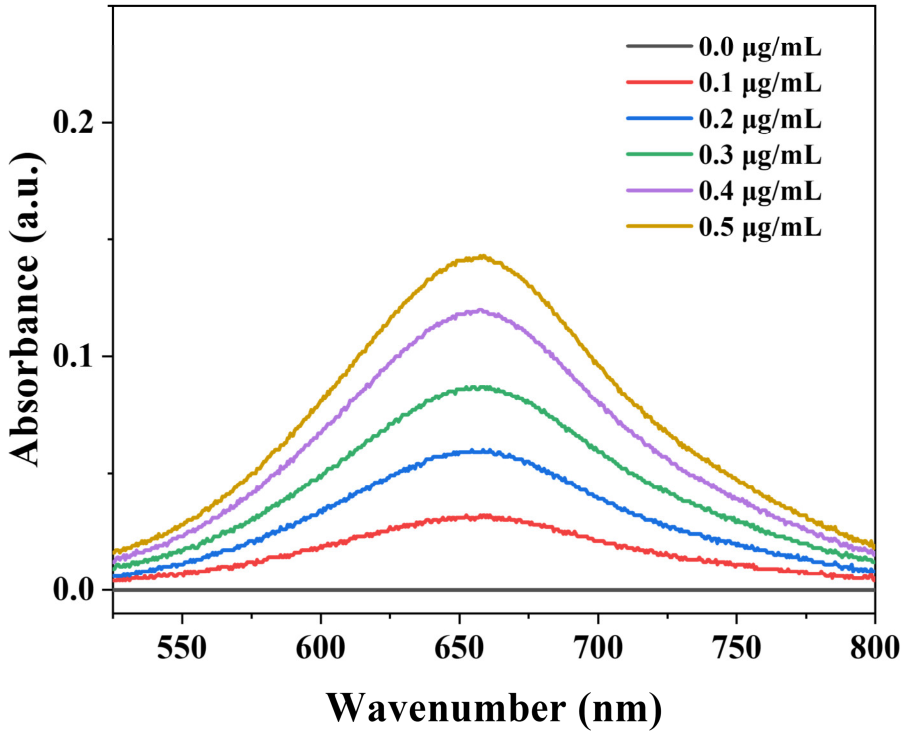


**Figure S15.** The UV-visible absorption curves corresponding to the coloration of indophenol blue after reaction with $NH_4^+$ of different concentrations.

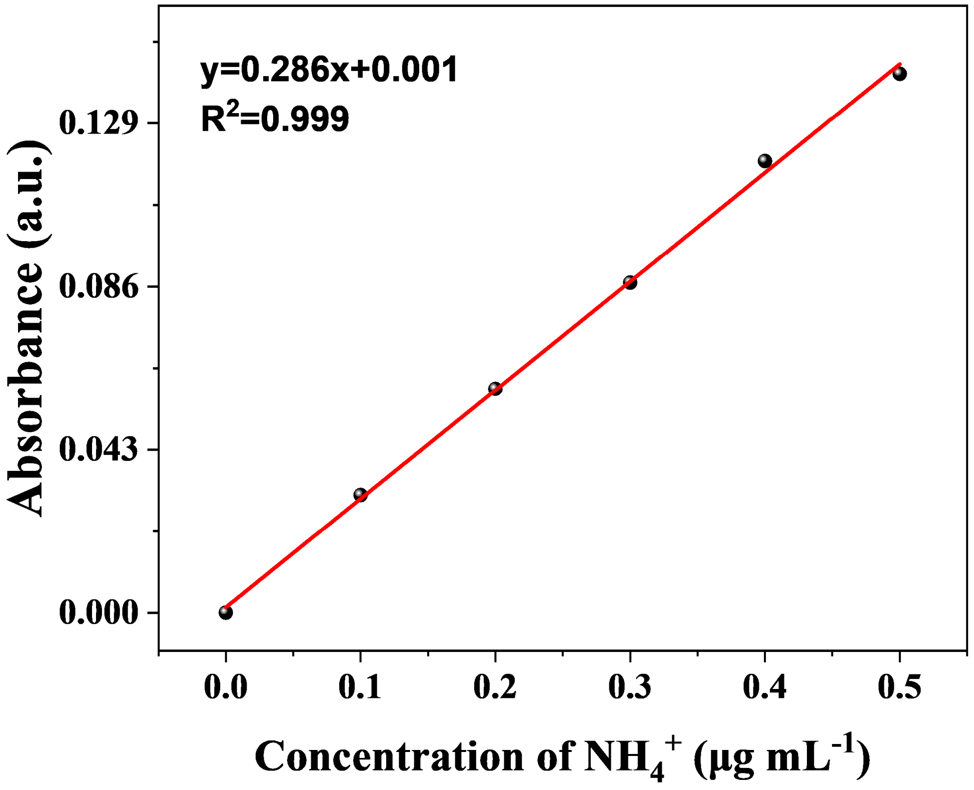


**Figure S16.** The linear fitting curves of absorbance at 655 nm after coloration by the indophenol blue method for $NH_4^+$ of different concentrations.

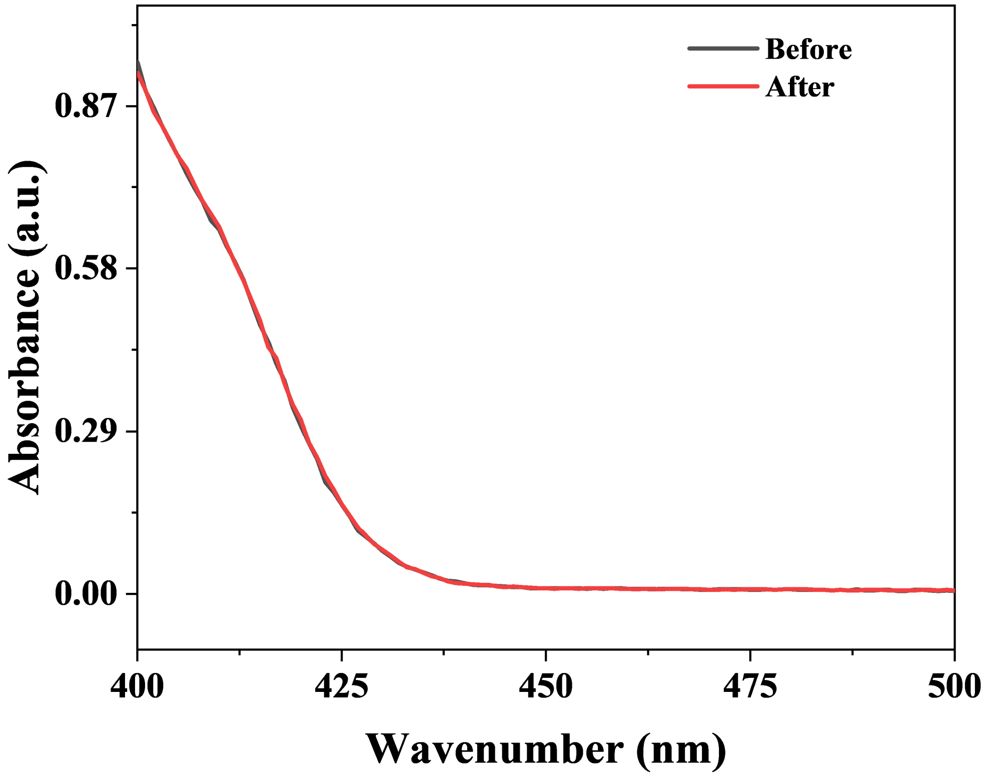


**Figure S17.** UV-Vis absorbance curves of Ru-TiNS before and after electrolysis at -0.3 V vs. RHE.

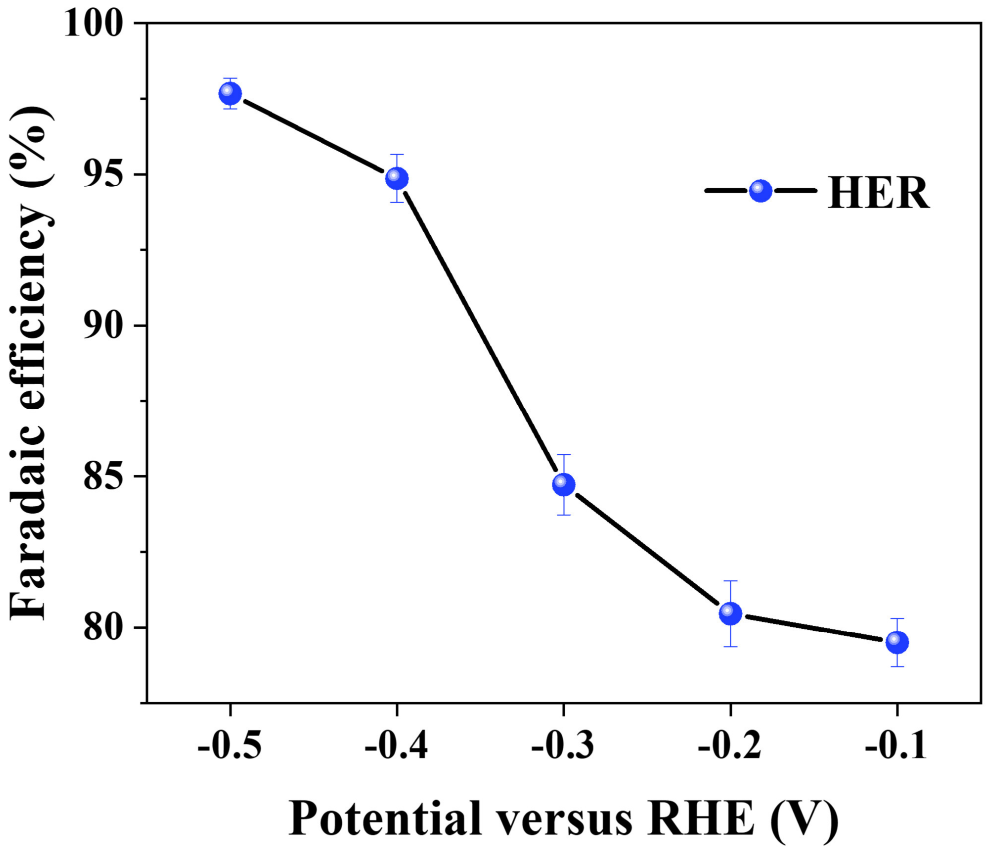


**Figure S18.** $FE_{H2}$ of Ru-TiNS at different potentials.

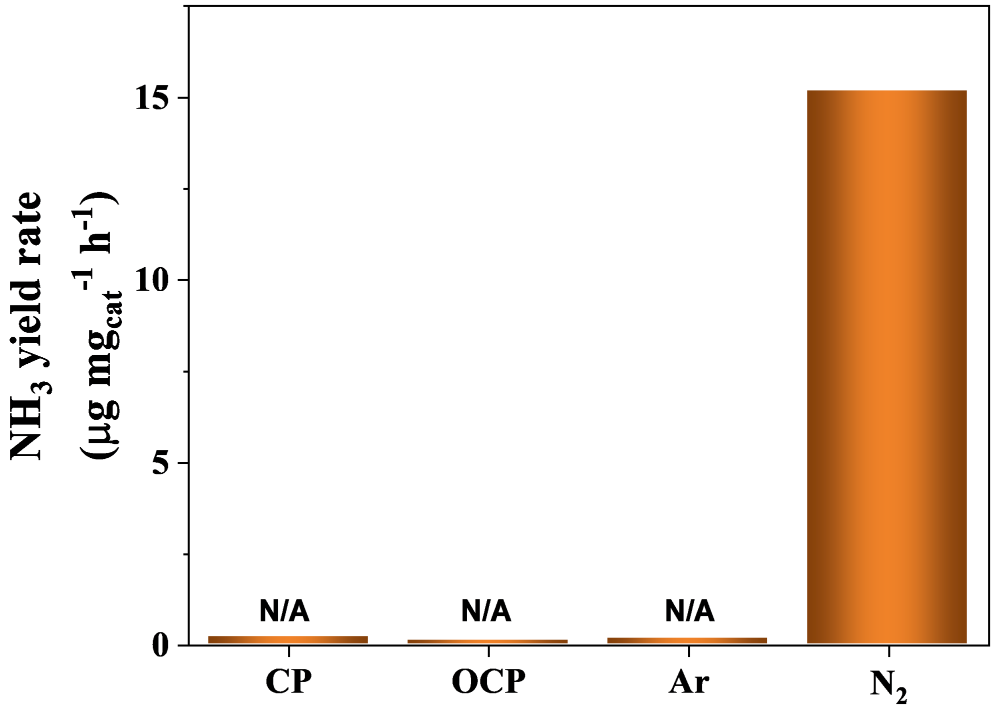


**Figure S19.** $NH_3$ yield rates under different conditions including pure carbon paper ( -0.3 V vs. RHE), open circuit potential, only Ar bubbling ( -0.3 V vs. RHE), and $N_2$ bubbling ( -0.3 V vs. RHE).

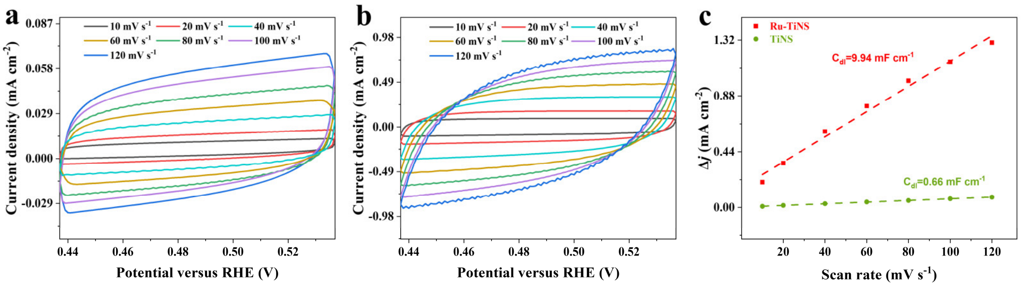


**Figure S20.** CV curves obtained for pure TiNS (a) and Ru-TiNS (b) at different scan rates, along with the corresponding calculated double-layer capacitances (c).

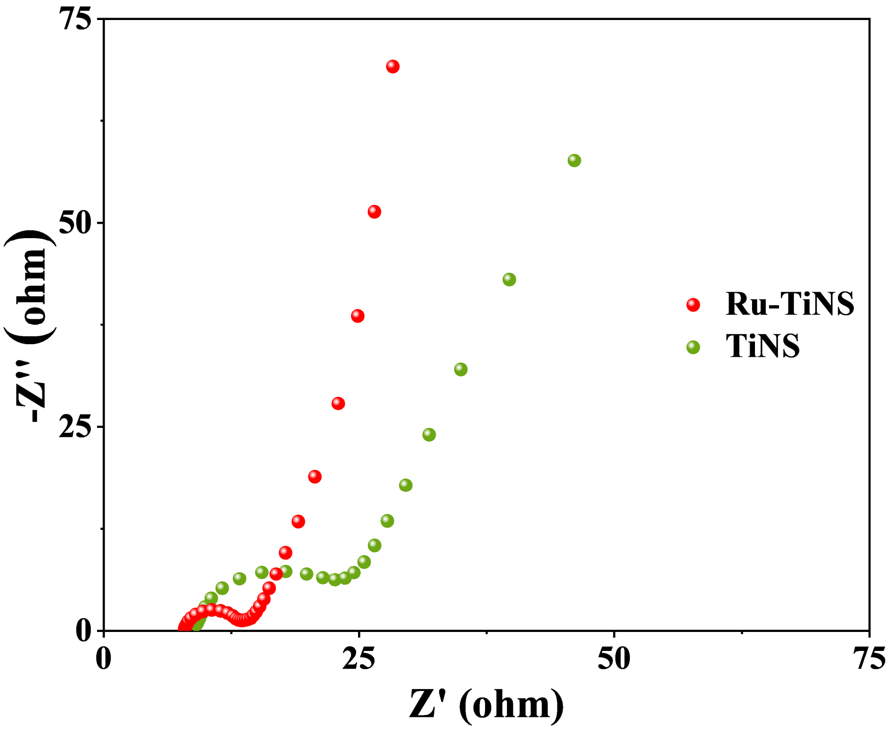


**Figure S21.** Electrochemical impedance spectroscopy (EIS) of Ru-TiNS and TiNS.

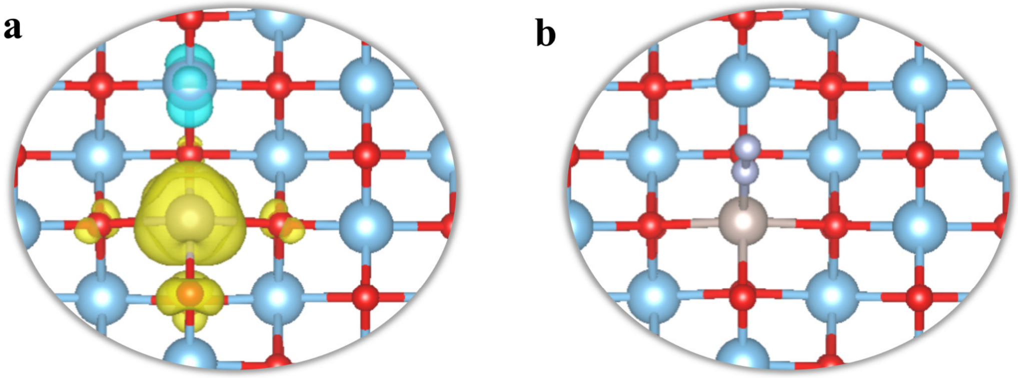


**Figure S22.** The Spin charge density of (a) Ru-TiNS and (b) $N_2$ adsorption on Ru-TiNS.

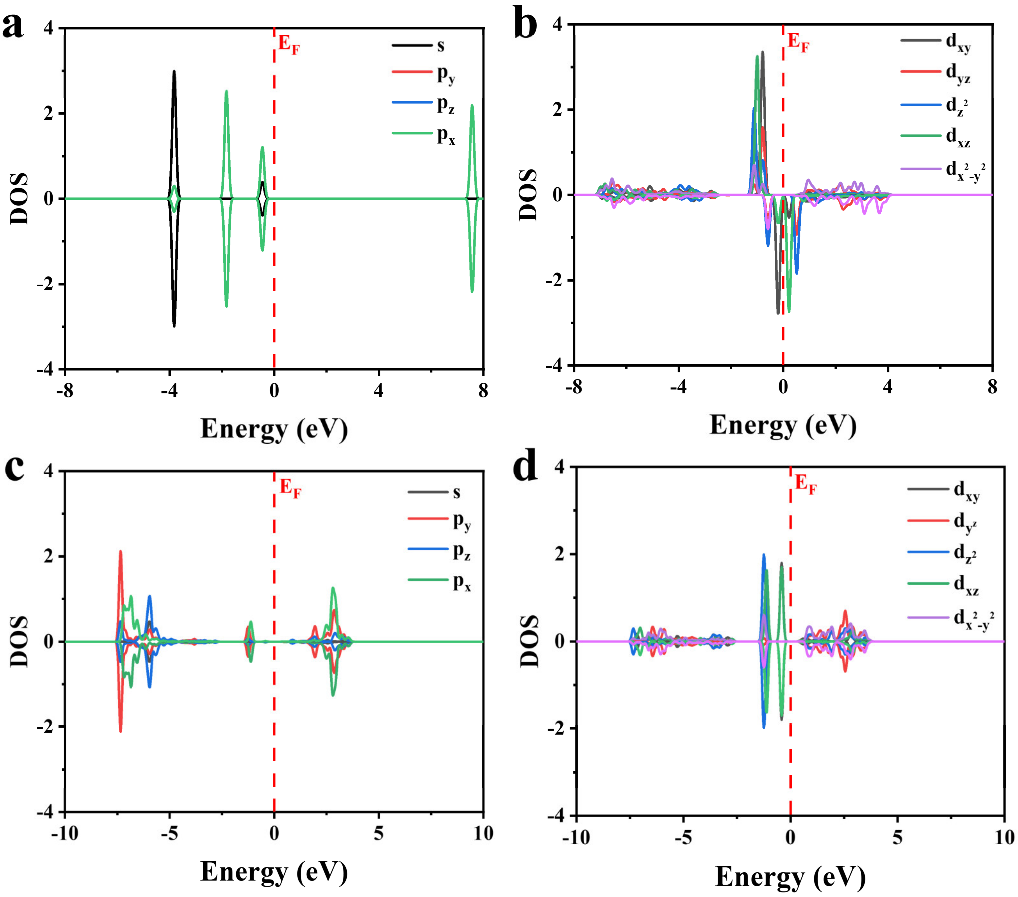


**Figure S23.** The projected density of (a) Free $N_2$, (b) Ru 4d in Ru-TiNS, and (c, d) $N_2$ molecule adsorbed on Ru-TiNS.
